# An Oxygenase-Independent Cholesterol Catabolic Pathway Operates under Oxic Conditions

**DOI:** 10.1371/journal.pone.0066675

**Published:** 2013-06-24

**Authors:** Po-Hsiang Wang, Tzong-Huei Lee, Wael Ismail, Ching-Yen Tsai, Ching-Wen Lin, Yu-Wen Tsai, Yin-Ru Chiang

**Affiliations:** 1 Biodiversity Research Center, Academia Sinica, Taipei, Taiwan; 2 Graduate Institute of Pharmacognosy, Taipei Medical University, Taipei, Taiwan; 3 Biotechnology Program, College of Graduate Studies, Arabian Gulf University, Manama, Kingdom of Bahrain; Institut Pasteur Paris, France

## Abstract

Cholesterol is one of the most ubiquitous compounds in nature. The 9,10-*seco*-pathway for the aerobic degradation of cholesterol was established thirty years ago. This pathway is characterized by the extensive use of oxygen and oxygenases for substrate activation and ring fission. The classical pathway was the only catabolic pathway adopted by all studies on cholesterol-degrading bacteria. *Sterolibacterium denitrificans* can degrade cholesterol regardless of the presence of oxygen. Here, we aerobically grew the model organism with ^13^C-labeled cholesterol, and substrate consumption and intermediate production were monitored over time. Based on the detected ^13^C-labeled intermediates, this study proposes an alternative cholesterol catabolic pathway. This alternative pathway differs from the classical 9,10-*seco*-pathway in numerous important aspects. First, substrate activation proceeds through anaerobic C-25 hydroxylation and subsequent isomerization to form 26-hydroxycholest-4-en-3-one. Second, after the side chain degradation, the resulting androgen intermediate is activated by adding water to the C-1/C-2 double bond. Third, the cleavage of the core ring structure starts at the A-ring via a hydrolytic mechanism. The ^18^O-incorporation experiments confirmed that water is the sole oxygen donor in this catabolic pathway.

## Introduction

Steroids are ubiquitous and structurally diverse in nature. Cholesterol is an essential structural component of animal cell membranes where it acts as a regulator of membrane fluidity and permeability. In addition, cholesterol serves as a crucial precursor for the biosynthesis of steroid hormones, bile acids, and vitamin D. Plants [1], [2] and fungi [3], [4] also synthesize small quantities of cholesterol. Although eukaryotes are the main producers of steroids, they lack degradation pathways for recycling the carbon content of these compounds. Hence, the degradation of steroids is dominated by bacteria [5]. Because steroids have limited functional groups, they are usually attacked by bacterial oxygenases using molecular oxygen as a co-substrate [6], [7].

The ubiquity and abundance of cholesterol renders the biodegradation of the C_27_ sterol a crucial issue in biogeochemistry. In previous years, the microbial transformation of steroids has attracted considerable attention because of its potential effects on biotechnological, pharmaceutical, and clinical applications [8], [9]. The investigation of cholesterol-degrading microorganisms began 70 years ago. In 1942, Tak observed that several *Mycobacterium* species could use cholesterol as their sole carbon and energy source [10]. Subsequent studies detected cholesterol-derived intermediates by growing various Gram-positive and Gram-negative bacteria with cholesterol [11]. The use of metabolic inhibitors such as α,α′, -dipyridyl (α,α′-D) enabled the significant accumulation of cholesterol-derived intermediates including androst-4-en-3,17-dione (AD) and androsta-1,4-diene-3,17-dione (ADD) [12]–[15].

**Figure 1:**
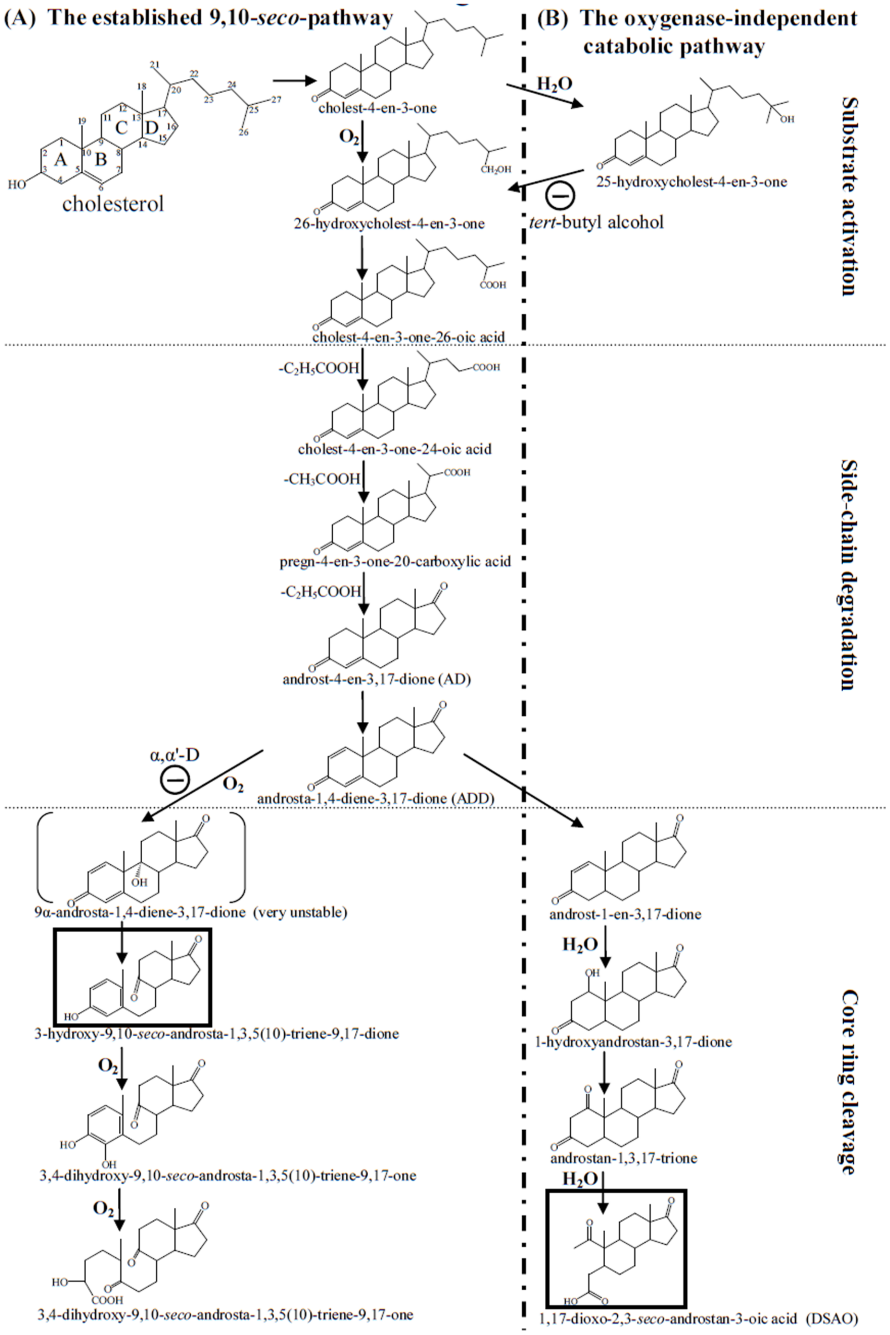
The aerobic catabolic pathways of cholesterol by bacteria. The ring identification (A–D) and carbon numbering systems (1–27) of steroids are shown in cholesterol. (A) The classical 9,10-*seco*-pathway demonstrated in *G. cholesterolivorans* DSMZ 45229. (B) The alternative 2,3-*seco*-pathway proposed in this study using *S. denitrificans* DSMZ 13999 as the model organism. 25-hydroxycholest-4-en-3-one was the last detected intermediate reported in the previous studies [32], [33]. First ring cleavage intermediates appearing in the catabolic pathways are highlighted in boxes. In this study, α,α′-D and *tert*-butyl alcohol served as the inhibitors for the 9,10-*seco*-pathway and 2,3-*seco*-pathway, respectively.

In the pioneering studies conducted by Sih et?al. [16], [17], the side-chain degradation of cholesterol by microbial activities was described. Sih et?al. [18]–[20] also established the mechanisms of oxygenolytic cleavage of steroidal rings. Kieslich then proposed a complete, oxygenase-dependent catabolic pathway for cholesterol in 1985 [6]. This pathway is characterized by the cleavage of the steroidal core ring between C-9 and C-10 (Figure 1A) and is called the 9,10-*seco*-pathway [21]. Following degradation of the aliphatic side-chain, several oxygenases cleave and degrade the core ring system of C_19_ steroid substrates. Introducing a hydroxyl group into ADD results in an extremely unstable intermediate, 9α-hydroxy-androsta-1,4-diene-3,17-dione. This compound thus undergoes simultaneous aromatization of the A-ring and cleavage of the B-ring (via a non-enzymatic reaction) to form 3-hydroxy-9,10-*seco*-androsta-1,3,5(10)-triene-9,17-dione. Further cleavage of the ring system proceeds through a hydroxylation at C-4. The aromatic A-ring then splits through the well-known *meta*-cleavage (Figure 1A). The aerobic testosterone catabolism of *Comamonas testosteroni* exhibits similar oxygenolytic ring cleavage mechanisms [7], [22]. The 9,10-*seco*-pathway is the only catabolic pathway for the microbial degradation of steroids described to date.

Recent studies have shown that the acquisition and catabolism of host cholesterol is a crucial process for the persistent infection of *Mycobacterium tuberculosis* in the lungs of chronically infected animals [23], [24]. Other studies have reported the purification and characterization of the key enzymes involved in the 9,10-*seco*-pathway [steroid C26-hydroxylase (CYP125) for substrate activation and 3-ketosteroid 9α-hydroxylase (KSH) for oxygenolytic core ring cleavage] from *M. tuberculosis* and its closely related strains [25]–[28].

A few reports have suggested the possibility of alternative catabolic pathways for the aerobic degradation of cholesterol [29], [30]. For example, the draft genome sequence of *Sterolibacterium denitrificans* DSMZ 13999 contains no steroid-transforming oxygenases [30]. This indirect evidence prompted us to study the aerobic cholesterol catabolism by the β-proteobacterium *S. denitrificans*, which is capable of growing aerobically and anaerobically with cholesterol using oxygen and nitrate as the terminal electron acceptors, respectively [31]. In a previous study, the initial steps of anaerobic cholesterol catabolism by *S. denitrificans* were investigated, and 25-hydroxycholest-4-en-3-one was the last detected intermediate [32] (for its structure, see Figure 1B). Very similar steps for substrate activation were suggested to occur in aerobic cholesterol catabolism by the same organism [33]. Recently, the molybdoenzyme of *S. denitrificans* that catalyzes catalyzing the anaerobic hydroxylation of the tertiary carbon (C-25) of C_27_ steroid substrates was purified and characterized [30].

Here, we adopted a ^13^C-metabolomic approach to detect the ^13^C-labeled intermediates involved in the aerobic cholesterol catabolism of *S. denitrificans*. Many detected intermediates are different from those of the classical 9,10-*seco*-pathway. Based on the ^13^C-metabolomics data and the time course data of cholesterol consumption and intermediates production, this study proposes an alternative cholesterol catabolic pathway, that does not require oxygenases for substrate activation and steroidal core ring cleavage (Figure 1B). The ^18^O-incorporation experiments conducted in this study confirm the O_2_-independent mechanisms.

## Results

### Cholesterol Catabolism by*S. denitrificans* is not Inhibited by α,α′-D

**Figure 2:**
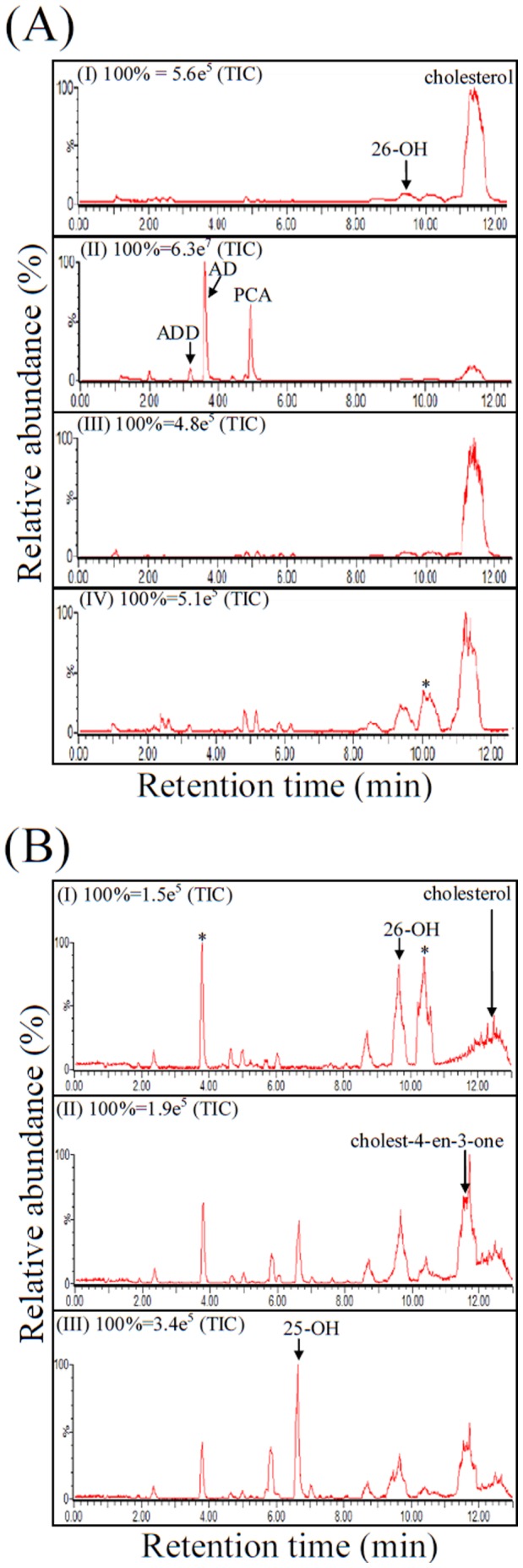
Aerobic cholesterol catabolic pathway by*S denitrificans* was inhibited by *tert*-butyl alcohol but not by α,α′-D. (A) UPLC-HRMS analysis of ethyl-acetate extracts of cholesterol-grown bacterial cells with or without α,α′-D. (AI) *G*. *cholesterolivorans* DSMZ 45229 grown with cholesterol (2 mM), (AII) *G*. *cholesterolivorans* grown with cholesterol and α,α′-D (5 mM), (AIII) *S. denitrificans* DSMZ 13999 grown with cholesterol, and (AIV) *S. denitrificans* grown with cholesterol and α,α′-D. (B) UPLC-HRMS analysis of ethyl-acetate extracts of cholesterol-grown *S. denitrificans* cells with different concentrations of *tert*-butyl alcohol. (BI) The aerobic growth without *tert*-butyl alcohol, (BII) in the presence of 2.5% (v/v) *tert*-butyl alcohol, and (BIII) in the presence of 5% *tert*-butyl alcohol. Abbreviations: TIC, total ion current; 26-OH, 26-hydroxycholest-4-en-3-one; 25-OH, 25-hydroxycholest-4-en-3-one; PCA, pregn-4-en-3-one-20-carboxylic acid; AD, androst-4-en-3,17-dione; ADD, androsta-1,4-diene-3,17-dione; *, unidentified nitrogen compounds.

To investigate the effect of α,α′-D on the cholesterol metabolism of *S. denitrificans*, we added α,α′-D (5 mM) to the culture after 1 mM cholesterol was consumed. *Gordonia cholesterolivorans* DSMZ 45229 was also tested for comparison. The addition of α,α′-D to the *G*. *cholesterolivorans* culture resulted in the accumulation of AD and ADD, indicating an interruption in the cholesterol catabolic pathway (Figure 2AII). The cholesterol-derived intermediates detected in the *G*. *cholesterolivorans* cultures were summarized in Table 1. HPLC analysis showed that two intermediates exhibited the characteristic maximal UV absorption at approximately 280 nm, indicating the presence of a phenolic A-ring (data not shown). These data indicated that *G*. *cholesterolivorans* uses the classical 9,10-*seco*-pathway to degrade cholesterol. On the contrary, α,α′-D did not inhibit the cholesterol degradation by *S. denitrificans* (Figure 2AIV).

**Table 1:** UPLC-HRMS and UV absorption behavior of the intermediates involved in aerobic cholesterol catabolism by*Gordonia cholesterolivorans* DSMZ 45229.

Compound ID	UPLC behavior (RT^a^,min)	Molecular formula/predicted molecular mass^b^	Dominant ion peaks	Identification of product ions	Mode observed	UV Absorption maximum
Cholesterol	11.72	C_27_H_46_O/386.3537	369.3531	[M-H_2_O+H]^+^	APCI and ESI	<210
Cholest-4-en-3-one	11.02	C_27_H_44_O/384.3381	385.3469 367.3365	[M+H]^+^ [M-H_2_O+H]^+^	APCI and ESI APCI and ESI	238
26-hydroxycholest-4-en-3-one	9.40	C_27_H_44_O_2_/400.3330	401.3431 383.3314	[M+H]^+^ [M-H_2_O+H]^+^	APCI and ESI APCI and ESI	241
Cholest-4-en-3-one-26-oic acid	7.39	C_27_H_42_O_3_/414.3123	415.3212 397.3107 379.3001 437.3065	[M+H]^+^ [M-H_2_O+H]^+^ [M-2H_2_O+H]^+^ [M+Na]^+^	APCI and ESI APCI and ESI APCI and ESI ESI	243
Cholest-4-en-3-one-24-oic acid	5.83	C_24_H_36_O_3_/372.2655	373.2743	[M+H]^+^	APCI and ESI	243
Pregn-4-en-3-one-20-carboxylic acid	4.94	C_22_H_32_O_3_/344.2343	345.2430 327.2324 309.2218	[M+H]^+^ [M-H_2_O+H]^+^ [M-2H_2_O+H]^+^	APCI and ESI APCI and ESI APCI	241
Androst-4-en-3,17-dione	3.62	C_19_H_26_O_2_/286.1926	287.2011 269.1905 309.1931	[M+H]^+^ [M-H_2_O+H]^+^ [M+Na]^+^	APCI and ESI APCI and ESI ESI	238
Androsta-1,4-diene-3,17-dione	3.18	C_19_H_24_O_2_/284.1770	285.1855 267.1749 307.1674	[M+H]^+^ [M-H_2_O+H]^+^ [M+Na]^+^	APCI and ESI APCI and ESI ESI	242

a RT, retention time.

b The predicated molecular mass was calculated using the atom mass of ^12^C (12.0000), ^16^O (15.9949), and ^1^H (1.0078).

Steroid C26-hydroxylase activity was detected in aerobically cholesterol-grown *G. cholesterolivorans* cells, but not in *S. denitrificans* cells (Table S1). These results suggested that *S. denitrificans* may adopt an alternative pathway to degrade cholesterol. This alternative pathway does not require monooxygenase-catalyzed hydroxylations at C-9 and C-26 of steroid substrates.

### *In vivo* Transformation of [4C-^13^C]Cholesterol by *S. denitrificans* Cells

**Figure 3:**
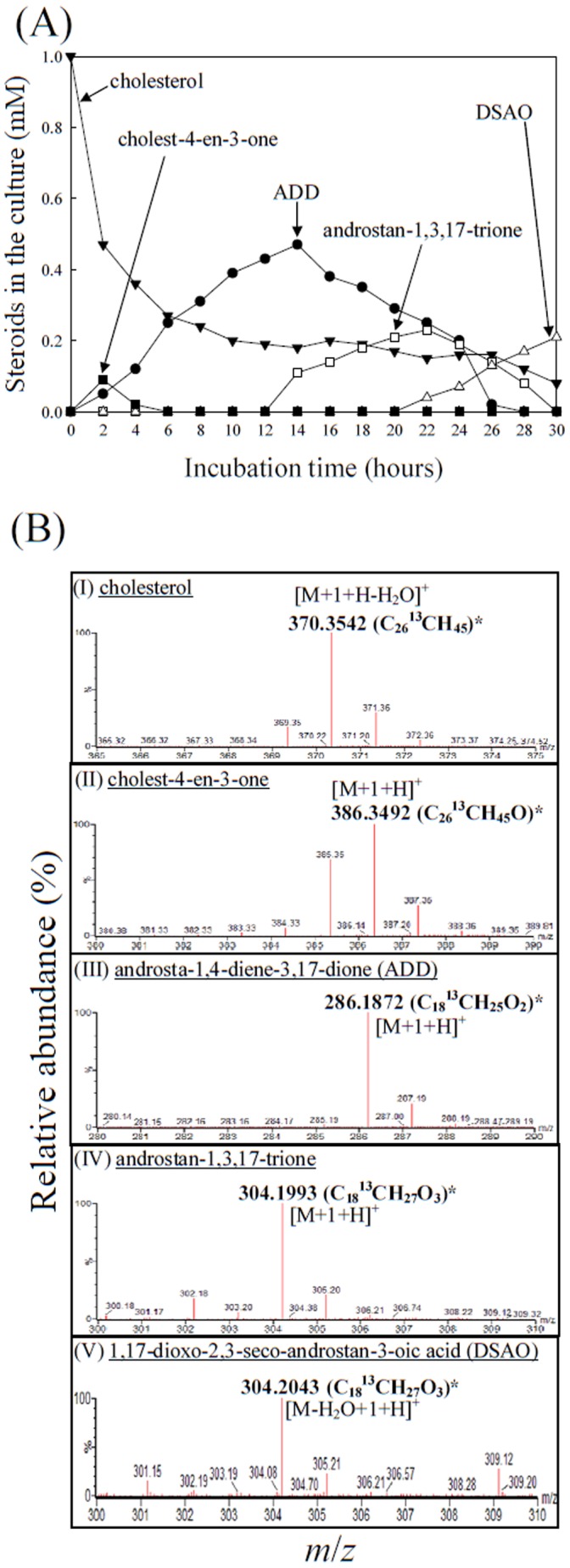
Aerobic cholesterol catabolism by*S. denitrificans* DSMZ 13999. (A) Time course of cholesterol consumption and intermediate production in a *S. denitrifican*s fed-batch culture. [4C-^13^C]cholesterol (1 mM) was fed to a starved *S. denitrificans* culture (OD_600nm_ = 0.9) after 2 mM cholesterol was exhausted. 0.1 mM of estrone was added as the internal control. The culture was incubation at 28°C for 30 hours with shaking (180 rpm). The steroids sampled at different time intervals were extracted with ethyl acetate three times, and the dominant intermediates were then separated and quantified using a reverse-phase HPLC system. Data are averages of three determinations (standard deviations <0.1). (B) High-resolution mass spectra of the dominant ^13^C-labeled intermediates detected in ethyl-acetate extracts of *S. denitrificans* cells grown on [4C-^13^C]cholesterol (1 mM). *The predicted elemental composition of individual intermediates was calculated using MassLynx™ Mass Spectrometry Software (Waters). See Figure S1 for other detected ^13^C-labeled intermediates derived from [4C-^13^C]cholesterol.

The *S. denitrificans* cells were grown with 1 mM [4C-^13^C]cholesterol. The time course of substrate consumption and intermediate production is shown in Figure 3A. The strong negative slope for cholest-4-en-3-one indicates that it is the first accumulated intermediate, which drastically decreased after 2 h of incubation. The strong positive slope for 1,17-dioxo-2,3-*seco*-androstan-3-oic acid (DSAO) indicates that it is the end product. The ADD and androstan-1,3,17-trione behaved like intermediates between cholest-4-en-3-one and DSAO. The ^13^C-labeled intermediates present in the ethyl acetate extracts were detected using ultra-performance liquid chromatography - high-resolution mass spectrometry (UPLC-HRMS), and their mass spectra are given in Figures 3B and S1. We detected a series of C_27_∼C_22_ acidic metabolites and C_19_ androgens. The acidic intermediates are the same as those shown in Table 1. These results indicate that in *S. denitrificans* cells, after the oxidation of the A-ring of cholesterol to form a 4-en-3-one structure, a series of β-oxidation and retro-aldol reactions degrade the aliphatic side-chain of cholesterol, as in the 9,10-*seco*-pathway. We also detected certain ^13^C-labeled intermediates that do not occur in the 9,10-*seco*-pathway (Figures 3BIV, 3BV, and S1A). One of them, identified as 1,17-dioxo-2,3-*seco*-androstan-3-oic acid by mass and NMR analyses, has an open A-ring structure.

In a previous *in vitro* study [32], 25-hydroxysteroids was transformed from cholest-4-en-3-one through an anaerobic hydroxylation mechanism. Here, we detected a tiny amount of ^13^C-labeled 25-hydroxycholest-4-en-3-one in the aerobically [4C-^13^C]cholesterol-grown *S. denitrificans* cells (Figure S1A). We then demonstrated that the presence of a competitive inhibitor, *tert*-butyl alcohol (5%, v/v), resulted in the apparent accumulation of this compound in aerobically cholesterol-grown *S. denitrificans* cells (Figure 2BIII). In contrast, in the absence of *tert*-butyl alcohol, 25-hydroxycholest-4-en-3-one did not accumulate (Figure 2BI). The dose-dependent result indicated that 25-hydroxycholest-4-en-3-one is a relevant intermediate of the alternative catabolic pathway. Note that the production of 26-hydroxycholest-4-en-3-one significantly decreased as *tert*-butyl alcohol was added to the aerobically cholesterol-grown *S. denitrificans* cells (Figure 2B). The data suggest that *tert*-butyl alcohol may inhibit the transformation of 25-hydroxycholest-4-en-3-one to 26-hydroxycholest-4-en-3-one.

### *In vivo* Transformation of [2,3,4C-^13^C]Testosterone by *S. denitrificans* Cells

*S. denitrificans* can aerobically grow with testosterone. In addition, C_19_ androgens were detected in the aerobically cholesterol-grown *S. denitrificans* cultures (Figures 3BIII, 3BIV, and 3BV). Therefore, we grew *S. denitrificans* with 1 mM of [2,3,4C-^13^C]testosterone in another 50 ml fed-batch culture to investigate the detailed C_19_ intermediates involved in this cholesterol catabolic pathway. The mass spectra of ^13^C-labeled intermediates derived from [2,3,4C-^13^C]testosterone are given in Figure S2. Seven C_19_ intermediates, including two acidic metabolites were observed. Four of these intermediates (1-testosterone, 1-hydroxyandrostan-3,17-dione, 17-hydroxy-1-oxo-2,3-*seco*-androstan-3-oic acid, and 1,17-dioxo-2,3-*seco*-androstan-3-oic acid) do not occur in the 9,10-*seco*-pathway (Figures S2E∼H). Moreover, we could not detect phenolic compounds (with a maximal UV absorption at approximately 280 nm) in the ethyl acetate extracts of *S. denitrificans*.

### Structural Elucidation of a Novel Cholesterol-derived Intermediate

**Figure 4:**
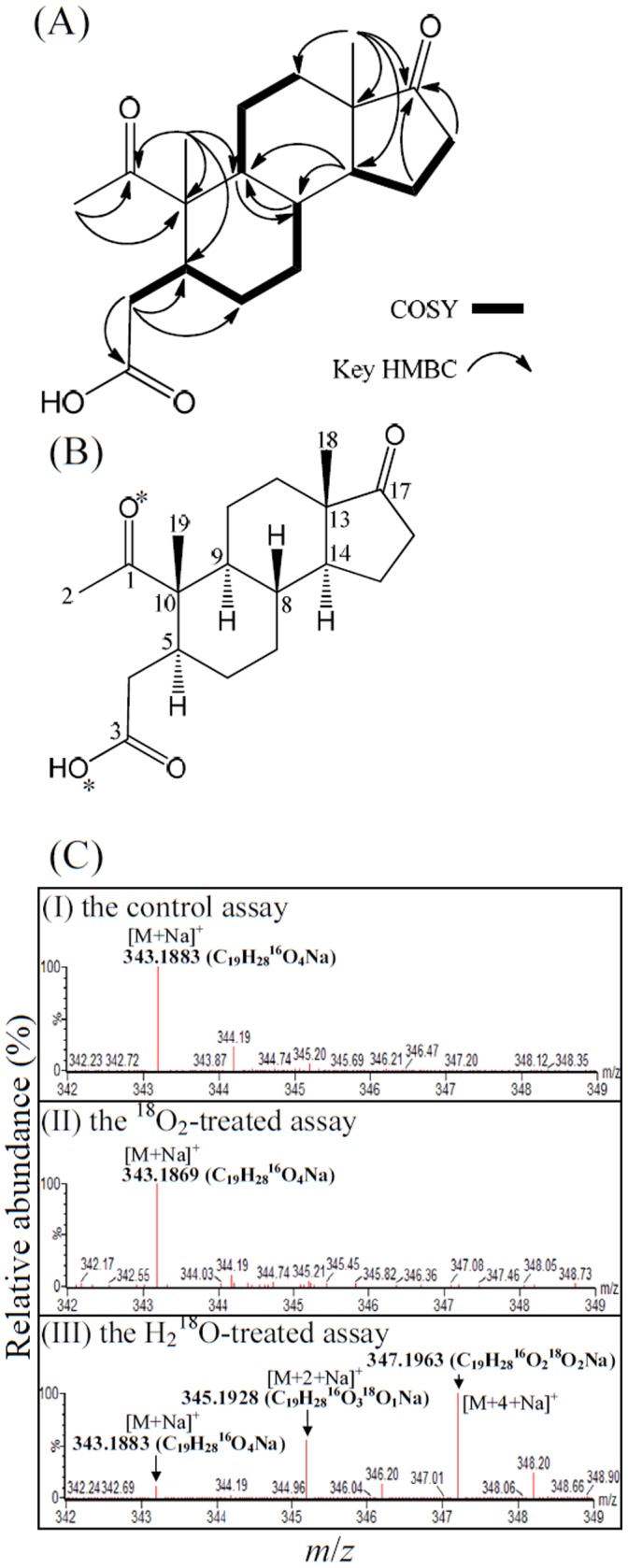
The structure elucidation and the investigation of the ring cleavage mechanism of compound 1 (1,17-dioxo-2,3-*seco*-androstan-3-oic acid, DSAO). (A) The interpretations of COSY and key HMBC spectra of compound **1.** (B) The chemical structure of compound **1**. *The oxygen atoms were labeled with ^18^O in the H_2_^18^O-incorporation assay. (C) ESI-mass spectra (positive ion mode) of DSAO. (CI) DSAO purified from the anaerobic control assay. (CII) DSAO purified from the ^18^O_2_-treated assay. (CIII) DSAO purified from the ^18^O-labeled H_2_O-treated assay. For detailed NMR spectral data of compound **1**, see Table S2.

To produce sufficient cholesterol-derived intermediates for NMR analysis, we grew four *S. denitrificans* (500 ml in 2 l Erlenmeyer flasks) cultures with unlabeled cholesterol (2 mM). After the consumption of 1.5 mM cholesterol, the cholesterol-derived intermediates were extracted with ethyl acetate. The separation of ethyl acetate extracts involved silica gel chromatography, TLC, and HPLC. Most cholesterol-derived intermediates (cholest-4-en-3-one, cholest-4-en-3-one-26-oic acid, pregn-4-en-one-20-carboxylic acid, AD, ADD, androst-1-en-3,17-dione, and 1-hydroxyandrostan-3,17-one) were identified by reference to the TLC, HPLC, UV absorption, and UPLC-HRMS behavior of authentic steroid standards. Compound **1**, an unprecedented cholesterol-derived intermediate, was isolated as a white powder. The structural elucidation of this compound relied mainly on mass and NMR spectra (see Figure S4 for original NMR spectra). The ESI-mass spectrum of compound **1** showed a sodium adduct ion of 343.1876 Da (Figure S3A). In addition, its pseudo-molecular ions [M –2H_2_O+H]^+^ and [M – H_2_O+H]^+^ at *m/z* 285.1862 and 303.1962, respectively, appeared in the APCI-mass spectrum (Figure S3B). Its molecular formula is thus deduced as C_19_H_28_O_4_. The ^13^C-NMR of compound **1** accompanied with DEPT interpretations showed signals including three methyl carbons, seven methylene carbons, four methine carbons, and five quaternary carbons (Table S2). The ^1^H NMR coupled with HSQC experiments exhibited signals for three primary methyls [δ_H_ 2.18 (3H), 0.99 (3H), and 0.87 (3H)], seven methylenes [δ_H_ 2.45, 2.08 (each 1H), 2.00, 1.60 (each 1H), 1.98, 1.86 (each 1H), 1.91, 1.17 (each 1H), 1.81, 1.35 (each 1H), 1.71, 1.28 (each 1H), and 1.47, 1.04 (each 1H)], and four methines [δ_H_ 2.36 (1H), 1.57 (1H), 1.55 (1H), and 1.42 (1H)] (Table S2). The connectivity of its C-8 and C-9 was approached by the HMBC spectrum (Figure S4D), in which the cross-peaks of H-8/C-9 and H-9/C-8 were observed. Together, the interpretations of distinctive cross-peaks in the COSY and HMBC spectra (Figure 4A) allowed the assignment of compound **1** as a 2,3-*seco*-androstane skeleton with one hydroxyl at C-3 and three ketone functionalities at C-1, C-3, and C-17, respectively. Accordingly, compound **1** was characterized as the shown structure, and named as 1,17-dioxo-2,3-*seco*-androstan-3-oic acid (Figure 4B).

### A Hydrolytic Ring Cleavage Mechanism Is Adopted by*S. denitrificans* to Degrade Cholesterol

In the classical 9,10-*seco*-pathway, oxygenases catalyze oxygenolytic ring fission using molecular oxygen as the co-substrate. To determine the origin of the oxygen atoms at C-1 and C-3 of 1,17-dioxo-2,3-seco-androstan-3-oic acid (DSAO), we conducted three *in?vitro* transformation assays using 1-testosterone (which has two ^16^O atoms at C-3 and C-17) as the substrate: (*i*) an H_2_^18^O-treated assay contained approximately 65% H_2_^18^O (mole/mole) in the reaction mixture, (*ii*) an ^18^O_2_-treated assay, and (*iii*) a control assay without the introduction of H_2_^18^O or ^18^O_2_. Similar to the DSAO purified from the control assay (Figure 4CI), no additional ^18^O-isotopic sodium adduct ion appeared in the ESI-mass spectrum of the DSAO purified from the ^18^O_2_-treated assay (Figure 4CII). In contrast, the ESI-mass spectrum of DSAO purified from the H_2_^18^O-treated assay showed three dominant sodium adduct ions ([M+Na]^+^, *m/z = *343.1883, 345.1928, and 347.1963; Figure 4CIII). Their elemental compositions were respectively calculated as C_19_H_28_^16^O_4_Na, C_19_H_28_^16^O_3_^18^ONa, and C_19_H_28_^16^O_2_^18^O_2_Na using MassLynx™ Mass Spectrometry Software (Waters). These data indicate that in the alternative pathway, after the activation of the A-ring through a hydration reaction, the cleavage of the core ring system of cholesterol begins with the A-ring by a hydrolysis reaction.

### 25-Hydroxycholest-4-en-3-one is Transformed to 26-Hydroxycholest-4-en-3-one in*S. denitrificans*

**Figure 5:**
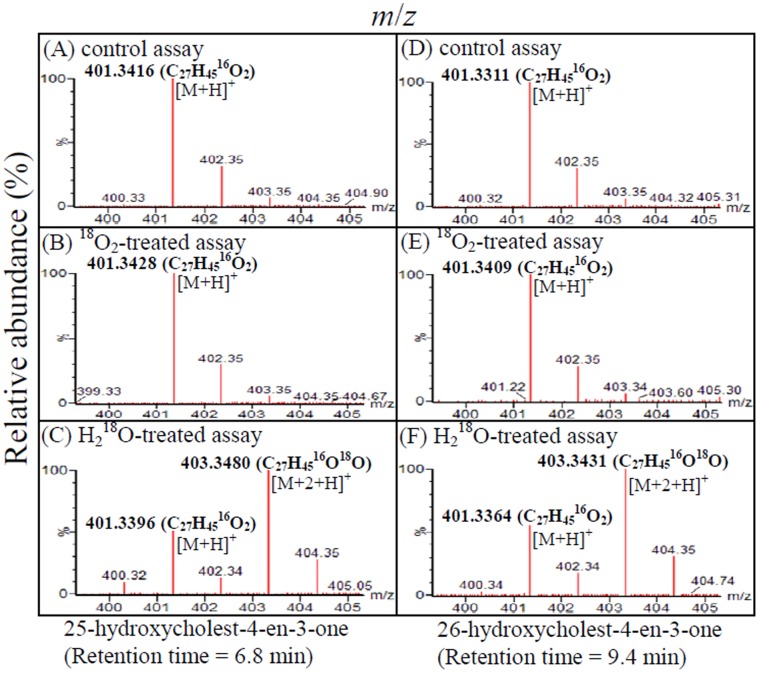
APCI-mass spectra (positive mode) of UPLC-separated 25-hydroxycholest-4-en-3-one (25-OH) and 26-hydroxycholest-4-en-3-one (26-OH). (A) 25-OH produced in the anoxic control assay. (B) 25-OH produced in the ^18^O_2_-treated assay. (C) 25-OH produced in the H_2_^18^O-treated assay. (D) 26-OH produced in the anoxic control assay. (E) 26-OH produced in the ^18^O_2_-treated assay. (F) 26-OH produced in the H_2_^18^O-treated assay.

In the 9,10-*seco*-pathway, 26-hydroxycholest-4-en-3-one is produced from cholest-4-en-3-one by steroid C26-hydroxylase, which requires oxygen and NADH as the co-substrate and the electron donor, respectively [28]. Steroid C26-hydroxylase activity was not detected in aerobically cholesterol-grown *S. denitrificans* cells (Table S1). In an ^18^O-labeled water-treated assay, the anaerobic reaction mixture (1 ml) containing ∼65% H_2_^18^O (mole/mole), soluble protein fraction precipitated at 25% ammonia sulfate saturation (1.2 mg), 5% (w/v) hydroxypropyl-β-cyclodextrin, K_3_[Fe(CN)_6_] (5 mM), and cholest-4-en-3-one (2 mM) was incubated for 16 h. The ethyl acetate-extractable sample was then analyzed using UPLC- atmospheric pressure chemical ionization (APCI)-HRMS. The two constitutional isomers (25-hydroxycholest-4-en-3-one and 26-hydroxycholest-4-en-3-one) can be distinguished easily because they exhibited different UPLC behavior (Figure 5). In the mass spectrum of 25-hydroxycholest-4-en-3-one, around two thirds of the product showed an ^18^O signal which originated from H_2_^18^O (Figure 5C). ^18^O-incorporation signal was also observed in 26-hydroxycholest-4-en-3-one produced from the ^18^O-labeled water-treated assay (Figure 5F). In contrast, no ^18^O-incorporation signals were detected in the hydroxylated steroids produced in the control assay (Figures 5A and 5D) and ^18^O_2_-treated assay (Figures 5B and 5E).

## Discussion

### An Alternative Cholesterol Catabolic Pathway is Present in*S. denitrificans*

In this study, we used ^13^C-labeled cholesterol as a tracer to identify the downstream C_27_∼C_19_ intermediates (from 26-hydroxycholest-4-en-3-one to DSAO, see Figure 1B for their structures) of the aerobic cholesterol catabolic pathway. According to the ^13^C-metabolomics and ^18^O-incorporation data, we demonstrate that *S. denitrificans* adopts an oxygenase-independent strategy to degrade cholesterol under oxic conditions. In the proposed pathway, 1,17-dioxo-2,3-*seco*-androstan-3-oic acid (the product of core ring cleavage) serves as the key intermediate. This compound has never been reported to be involved in aerobic steroid catabolism. Since the 9,10-*seco*-pathway was established thirty years ago [6], this is the first documented case that clearly demonstrates the existence of an alternative cholesterol catabolic pathway in bacteria. The proposed alternative pathway is significantly different from the 9,10-*seco*-pathway.

### Comparison with the Classical 9,10-*seco*-pathway

In the alternative catabolic pathway, the side-chain degradation also precedes the core ring cleavage (Figure 1B). However, the alternative pathway differs from the established 9,10-*seco*-pathway in the mechanisms of substrate activation and core ring cleavage. In the *in?vivo* [4C-^13^C]cholesterol biotransformation assay, both ^13^C-labeled 25-hydroxycholest-4-en-3-one and 26-hydroxycholest-4-en-3-one could be detected. The NADH-dependent steroid C26-hydroxylase was purified from *Rhodococcus jostii* [28] and *Mycobacterium tuberculosis* [34]. However, neither the corresponding gene [30] nor the enzyme activity (this study) of steroid C26-hydroxylase could be detected in *S. denitrificans* cells. Moreover, we confirmed that the terminal hydroxyl group of 26-hydroxycholest-4-en-3-one produced by *S. denitrificans* originates from water. These data indicate that in the aerobic cholesterol catabolism by *S. denitrificans*, 26-hydroxycholest-4-en-3-one may be produced from 25-hydroxycholest-4-en-3-one through a novel isomerization reaction. Similar isomerization reactions of hydroxyl groups occur in monoterpene metabolism [35], [36]. Researchers have recently isolated and characterized linalool dehydratase-isomerase [37]. This enzyme catalyzes the migration of a hydroxyl group from a tertiary carbon to a primary one. A similar enzyme might catalyze the isomerization of 25-hydroxycholest-4-en-3-one to a 26-hydroxyl structure.

After substrate activation by adding a hydroxyl group at C-26, the aliphatic side-chain of the C_27_ steroid substrates is degraded through a series of retro-aldol and β-oxidation reactions to form C_24_, C_22_ acidic intermediates and C_19_ androgens, which were observed in cholesterol-grown *S. denitrificans* cultures. These side-chain-degrading reactions do not require oxygenases. In contrast to the 9,10-*seco*-pathway, the proposed catabolic route applies no oxygenases for ring fission (Figure 1B). The core ring structure opens first at the A-ring through a hydrolytic mechanism. The ^18^O-incorporation experiments corroborate the proposed hydrolytic ring cleavage mechanism.

Recently, new pathways for the degradation of aromatic compounds under oxic conditions were unraveled [38], [39]. These pathways operate primarily in facultative anaerobes and use a hydrolytic mechanism to open the ring of the substrates. However, these aerobic pathways still employ monooxygenases to introduce hydroxyl groups into the aromatic ring for substrate activation. In contrast, the proposed aerobic cholesterol degradation pathway does not require any oxygenases-catalyzed reactions till the stage at which the steroidal A-ring opens.

### Potential Ecological Significance of the Proposed Cholesterol Degradation Pathway

*S. denitrificans* can degrade cholesterol regardless of the presence of oxygen. Several lines of evidence suggest that very similar metabolic strategies may be adopted by *S. denitrficans* to degrade cholesterol under oxic and anoxic conditions. First, previous proteome analyses revealed no apparent differences in soluble protein patterns of anaerobically and aerobically grown *S. denitrificans* cells [33]. Second, the steroid-transforming enzymes involved in the initial steps of anaerobic cholesterol metabolism by *S. denitrificans* are not oxygen-labile *in?vivo* [30], [32]. Third, the O_2_-dependent steroid-transforming enzymes, including steroid C26-hydroxylase and 3-ketosteroid 9α-hydroxylase, are not detected in aerobically cholesterol-grown *S. denitrificans* cells (this study).

It is tempting to speculate that *S. denitrificans* have developed efficient mechanisms to profit from the available carbon sources regardless of the prevailing redox state. The first adaptive mechanism could be the ability to initiate the degradation of steroid substrates under both oxic and anoxic conditions via similar reactions and intermediates [33]. The second mechanism involves the adoption of oxygenase-independent aerobic catabolic pathways. Both mechanisms would enhance the metabolic competence of these organisms because they can switch quickly between aerobic and anaerobic metabolic modes. Moreover, under micro-aerobic conditions, when the oxygen tension becomes insufficient, the organisms can channel the oxygen flux to the respiratory electron transport chain, and still profit from the steroid substrate through oxygenase-independent catabolic pathways that do not consume molecular oxygen.

Recently, very similar ring cleavage mechanisms were observed in anaerobic testosterone degradation by a γ-proteobacterium, *Steroidobacter denitrificans* DSMZ 18526 [40]. Interestingly, the bacterial strain uses the 9,10-*seco*-pathway to degrade testosterone when oxygen is available. These data indicated that bacteria adopt the oxygenase-independent 2,3-*seco*-pathway to degrade steroids not only under anaerobic conditions [40], but also under aerobic conditions (at least in this case). So far, less is known about the enzymes (especially the A-ring-cleavage enzyme) and their corresponding genes involved in the 2,3-*seco*-pathway. Therefore, *in situ* ^13^C-metabolomics seems to be a feasible approach to investigate the contribution of the 2,3-seco-pathway in the degradation of cholesterol and other steroids in natural environments and engineered systems.

### Conclusions

The results of this study demonstrate that microbial degradation of one substrate can proceed via different mechanisms under the same conditions. The cholesterol degradation pathway proposed in this study further underpins the diversity of microbial catabolism of organic compounds. It also broadens our understanding of the strategies that microorganisms use to cope with and adapt to environmental conditions and challenging inert substrates such as steroids.

## Materials and Methods

### Chemicals and Bacterial Strains

The [4C-^13^C]cholesterol, ^18^O-labeled water (97 atom%), and ^18^O_2_ (99 atom%) were purchased from Sigma-Aldrich. [2,3,4C-^13^C]testosterone was obtained from Isosciences. The chemicals were of analytical grade and were purchased from Fluka, Mallinckrodt Baker, Merck, or Sigma-Aldrich. *Sterolibacterium denitrificans* DSMZ 13999 and *Gordonia cholesterolivorans* DSMZ 45229 were obtained from the Deutsche Sammlung für Mikroorganismen und Zellkulturen (Braunschweig, Germany).

### The Preparation of Steroid Intermediates

25-Hydroxycholest-4-en-3-one was produced *in?vitro* and purified as described elsewhere [32]. 1-Testosterone, androst-1-en-3,17-dione, and 1-hydroxysteroids (C_19_) were produced and purified as mentioned [41].

### Fed-batch Growth of*S. denitrificans* with Unlabeled Cholesterol

In this study, 0.5% of hydroxypropyl-β-cyclodextrin was always added to the bacterial cultures to improve the solubility of cholesterol in media. *S. denitrificans* was grown in phosphate-buffered shake-flask cultures (500 ml in 2 l Erlenmeyer flasks) containing 2 mM cholesterol. The culture was incubated at 28°C in an orbital shaker (180 rpm). In 1 l of distilled water, the medium contained the following: 0.77 g cholesterol, 5 g hydroxypropyl-β-cyclodextrin, 1.0 g NH_4_Cl, 0.5 g MgSO_4_⋅7 H_2_O, and 0.1 g CaCl_2_⋅2H_2_O. After autoclaving, sterile 50 ml KH_2_PO_4_-K_2_HPO_4_ buffer solution (1 M, pH 7.0), vitamins (1 ml l^−1^) [42], EDTA-chelated mixture of trace elements (1 ml l^−1^) [43], and selenite and tungstate solution (1 ml l^−1^) [44] were added. The amounts of residual cholesterol in the cultures were monitored using HPLC. After the consumption of 1.5 mM cholesterol, the pH of the cultures was adjusted to pH <2 using 5M HCl. The acid-treated cultures were extracted 3 times with the same volume of ethyl acetate to recover the residual cholesterol and its derivatives from the aqueous phase. The separation of ethyl acetate extracts was performed using silica gel chromatography, TLC, and HPLC.

### Cholesterol Catabolism by*S. denitrificans* in the Presence of α,α′-D

*S. denitrificans* was grown in two phosphate-buffered shake-flask cultures (50 ml in 250 ml-Erlenmeyer flasks) containing 2 mM cholesterol at 28°C with shaking. After the consumption of 1 mM cholesterol, 5 mM of α,α′-D (an inhibitor of 3-ketosteroid 9α-hydroxylase [12]–[15]) was added to one of the culture, and the incubation of both cultures continued for 16 h. The pH of the cultures was subsequently adjusted to pH <2, and ethyl acetate was used to extract cholesterol-derived neutral and acidic intermediates. Cholesterol metabolism by *G. cholesterolivorans* DSMZ 45229 was also studied using the same procedure for comparison. The four ethyl acetate extracts were analyzed using UPLC-HRMS.

### Effect of*tert*-Butyl Alcohol on Cholesterol Catabolism of *S. denitrificans*

*S. denitrificans* was aerobically grown in three phosphate-buffered shake-flask cultures (50 ml) containing 2.5 mM cholesterol. After the consumption of 2 mM cholesterol, 2.5% and 5% (v/v) *tert*-butyl alcohol (an analog of 25-hydroxycholest-4-en-3-one) was individually added to two cultures. 2-Propanol was then added to three cultures to bring the final alcohol concentration to 5% (v/v) in all cultures. The incubation of the three cultures continued further 16 h. The pH of the cultures was adjusted to pH <2, and ethyl acetate was used to extract cholesterol-derived intermediates. The three ethyl acetate extracts were analyzed using UPLC-HRMS.

### Fed-batch Growth of*S. denitrificans* with [4C-^13^C]Cholesterol

A *S. denitrificans* culture (500 ml) was first grown with 2 mM of unlabeled cholesterol in a 2 l Erlenmeyer flask. After the unlabeled cholesterol was completely consumed, 50 ml of the stock culture was transferred into a sterile 250-ml Erlenmeyer flask. The *S. denitrificans* cells were subsequently fed with 1 mM [4C-^13^C]cholesterol and incubated at 28°C with shaking (180 rpm). Estrone (0.1 mM) which cannot be utilized by *S. denitrificans* as a carbon and energy source was added as an internal control. Samples (3 ml) were withdrawn every two hours. Culture samples (0.1 ml ×3) were centrifuged at 10,000×*g* for 10 min to harvest the *S. denitrificans* cells. The protein content in the pellet was determined using bicinchoninic acid (BCA) assay. The residual culture samples (2.7 ml) were acidified to pH <2, and extracted three times with the same volume of ethyl acetate to recover cholesterol-derived intermediates. The ethyl acetate fractions were combined, the solvent was evaporated, and the residue was re-dissolved in 300 µl of methanol. The [4C-^13^C]cholesterol-derived intermediates in 60 µl samples were identified using ultra-performance liquid chromatography - high-resolution mass spectrometry (UPLC-HRMS). The amount of residual cholesterol and cholesterol-derived intermediates in the samples (80 µl ×3) was determined using HPLC.

### Fed-batch Growth of*S. denitrificans* with [2,3,4C-^13^C]Testosterone

In another 50 ml *in?vivo* biotransformation assay, *S. denitrificans* (50 ml culture in a 250-ml Erlenmeyer flask) transferred from the same stock culture was fed with 1 mM [2,3,4C-^13^C]testosterone. The samples (3 ml) were withdrawn after 16h incubation. The pH of the culture samples was adjusted to pH <2 using 5M HCl. The ethyl acetate -extractable samples were analyzed using UPLC-HRMS.

### Preparation of Cell Extracts

The *S. denitrificans* cultures (500 ml in 2 l Erlenmeyer flasks) were grown with 2 mM cholesterol with shaking (180 rpm). Cells were harvested by centrifugation in the exponential growth phase at OD_600_ of 0.8∼1.0 (optical path 1 cm) and the cell pellet was then stored at −80°C. All steps used for preparation of cell extracts were performed at 4°C. Frozen cells were suspended in twice the volume of 150 mM Tris-HCl buffer (pH 7) containing 0.1 mg of DNase I ml^−1^. Cells were broken by passing the cell suspension through a French pressure cell (Thermo Fisher Scientific) twice at 137 MPa. The cell lysate was fractionated using two steps of centrifugation: the first step involved centrifugation for 30 min at 20,000×*g* to remove the cell debris, unbroken cells and residual cholesterol. The supernatant (crude cell extract) was then centrifuged at 100,000×*g* for 1.5 h to separate soluble proteins from membrane-bound proteins.

### *In vitro* ^18^O-Incorporation Assays for the Production of 1,17-Dioxo-2,3-*seco*-androstan-3-oic Acid

To determine the origins of the oxygen atoms at C-1 and C-3 of 1,17-dioxo-2,3-*seco*-androstan-3-oic acid, three *in?vitro* assays were performed in an anaerobic chamber containing 95% N_2_ and 5% H_2_ (1atm). The three reaction mixtures (3 ml for each assay) were incubated at 30°C for 16 h with shaking. After the acidic treatment, the steroid products were extracted from the assays using ethyl acetate, and the extracts were analyzed using UPLC-APCI-mass spectrometry.

#### (1) Control assay

A 3-ml reaction mixture containing 50 mM Tris-HCl buffer (pH 7) and soluble proteins (15 mg) of *S. denitrificans* were sealed in a 10-ml glass bottle with a rubber stopper. The reaction was started by adding 200 µl of 67.5 mM 1-testosterone solution (in 2-propanol) to the assay. The final concentration of the steroid substrate in the reaction mixture was 4.5 mM. The final 2-propanol content was 6.67%.

#### (2) 18O2-Treated assay

1.8 ml of ^18^O_2_ gas (99 atom %) was introduced into an anaerobic glass bottle containing 7 ml of headspace (95% N_2_ and 5% H_2_, 1atm) and 3 ml reaction mixture containing the same components as the control assay. The final^ 18^O_2_ concentration in the headspace was ∼20%.

#### (3) 18O-Labeled Water-treated assay

A 2.0 ml sample of^ 18^O-labeled water (97 atom %) was added to 1.0 ml of 150 mM Tris-HCl buffer (pH 7) containing soluble proteins of *S. denitrificans* (15 mg). The final ^18^O-water content was approximately 64.7%. The reaction was started by adding 4.5 mM of 1-testosterone to the anoxic assay. The 2-propanol content was also 6.67%.

### Activity Assays for Steroid C26-Hydroxylase (Cyp125)

The Cyp125 activity of *G. cholesterolivorans* and *S. denitrificans* was measured by monitoring the product (26-hydroxycholest-4-en-3-one) concentration using a Hitachi HPLC module. The reaction mixture (1 ml) contained an air-saturated 100 mM potassium phosphate buffer (pH 7.0), 5 mM NADH, 0.5 mM cholest-4-en-3-one, 5% (w/v) 2-hydroxypropyl-β-cyclodextrin, and soluble proteins (20 mg) precipitated at 50% ammonium sulfate saturation. In the anaerobic assays, 2 mM 1,4-dithiothreitol was added to remove residual O_2_ present in the reaction mixture (1 ml), which was prepared in an anaerobic chamber containing 95% N_2_ and 5% H_2_ (1atm). The aerobic and anaerobic assays were incubated at 30°C for 16 h with shaking. The reaction was stopped by the addition of 20 µl of 25% HCl, and the steroids were extracted using ethyl acetate.

### *In vitro* ^18^O-Incorporation Assays for the Production of 26-Hydroxycholest-4-en-3-one

To determine the origins of the oxygen atoms at C-26 of 26-hydroxycholest-4-en-3-one, three *in?vitro* assays were performed in an anaerobic chamber. In all assays, 20 µl of 100 mM cholest-4-en-3-one solution (in 2-propanol) was added to empty glass bottles (3-ml). After complete evaporation of the solvent, the reaction mixture (1 ml) was dispensed anaerobically. The three reaction mixtures (1 ml for each assay) were incubated at 30°C for 16 h with shaking. The ethyl acetate extracts were analyzed using UPLC-APCI-mass spectrometry.

1. *Control Assay.* In a 3-ml glass bottle sealed with a rubber stopper, the 1-ml reaction mixture contained 50 mM Tris-HCl buffer (pH 8), 2 mM cholest-4-en-3-one (pre-coated on the bottle wall), 5 mM K_3_[Fe(CN)_6_], 5% (w/v) hydroxypropyl-β-cyclodextrin, and soluble *S. denitrificans* proteins (1.2 mg) precipitated at 25% ammonia sulfate saturation.
2. *^18^O_2_-Treated Assay.* 0.5 ml of ^18^O_2_ gas (99 atom %) was introduced into an anaerobic glass bottle containing 2 ml of headspace (95% N_2_ and 5% H_2_, 1atm) and 1 ml reaction mixture containing the same components as the control assay. The final^ 18^O_2_ concentration in the headspace was ∼20%.
3. *^18^O-Labeled Water-Treated Assay.* A total of 0.67 ml of^ 18^O-labeled water (97 atom %) was added to 0.33 ml of 150 mM Tris-HCl buffer (pH 8) containing soluble proteins (1.2 mg) precipitated at 25% ammonia sulfate saturation, 15 mM K_3_[Fe(CN)_6_], 15% (w/v) hydroxypropyl-β-cyclodextrin. The final ^18^O-water content was approximately 65%. 2 mM (the final concentration) of cholest-4-en-3-one was pre-coated on the bottle wall as mentioned.

### Silica Gel Chromatography

A 385 ml silica gel column (55×3 cm; SiliaFlash® P60, Silicycle) was equilibrated with 2 bed volumes of *n*-hexane - ethyl acetate (65∶35, v/v). The ethyl acetate extract (approximately 400 mg dissolved in 3 ml ethyl acetate) containing cholesterol-derived intermediates was loaded into the column and eluted with the equilibrium solvent system at a flow rate of 2 ml min^−1^. The eluate was collected in 5-ml fractions, and a 0.5 ml sample was taken from each fraction. The solvent was evaporated to dryness, and the residue was re-dissolved in 10 µl of methanol. The samples were analyzed using TLC. The fractions containing the same compounds were pooled and evaporated to dryness, and 200 µl of methanol was used to dissolve the residue. Further purification of cholesterol-derived intermediates was performed using TLC.

### Thin Layer Chromatography (TLC)

The steroid products were then separated on silica gel aluminum TLC plates (ALUGRAM® Xtra SIL G/UVF_254_, thickness, 0.2 mm, 20×20 cm; Macherey-Nagel) using the following developing solvent system: dichloromethane - ethyl acetate - methanol (14∶4:1, v/v). The steroid compounds were visualized under UV light at 254 nm or by spraying the TLC plates with 30% (v/v) H_2_SO_4_.

### High-Performance Liquid Chromatography (HPLC)

A reversed-phase Hitachi HPLC system was used for the final separation. The separation was achieved on an analytical RP-C_18_ column (Luna 18(2), 5 µm, 150×4.6 mm; Phenomenex) incubated at 35°C. The mobile phase included a mixture of two solvents: A (0.1% aqueous trifluoroacetic acid) and B (methanol containing 0.1% trifluoroacetic acid). The C_27_ steroids were eluted at a flow rate of 1.0 ml/min with a gradient from 80%–90% B over 5 min, followed by isocratic elution at 90% B for 10 min, a gradient from 90%–100% B for 5 min, and further isocratic elution for 20 min. The separation of C_19_ steroids were performed at a flow rate of 1.0 ml/min with a gradient from 40%–65% B within 50 min. The steroid products were detected in the range of 200–300 nm using a photodiode array detector. The structures of HPLC-purified intermediates were elucidated using NMR spectroscopy and mass spectrometry. In addition, HPLC was used for the quantification of some steroid substrates and intermediates present in the *S. denitrificans* cultures. The quantification of steroids (cholesterol, cholest-4-en-3-one, ADD, androstan-1,3,17-trione, and 1,17-dioxo-2,3-*seco*-androstan-3-oic acid) was calculated from their respective peak areas using a standard curve of individual standards. The *R* ^2^ values for the standard curves were >0.98. Data are averages of three measurements.

### Ultra-Performance Liquid Chromatography–Atmospheric Pressure Chemical Ionization–High-Resolution Mass Spectrometry (UPLC-APCI-HRMS)

The ethyl acetate extractable samples or HPLC-purified steroid intermediates were analyzed using UPLC-MS with UPLC coupled to an APCI-mass spectrometer. Mass spectral data were obtained using a Waters HDMS-QTOF synapt mass spectrometer (Waters) equipped with a standard APCI source operating in the positive ion mode. Separation was achieved on a reversed-phase C_18_ column (Acquity UPLC® BEH C18, 1.7 µm, 100×2.1 mm; Waters) with a flow rate of 0.3 ml min^−1 ^at 50°C (column oven temperature). The mobile phase comprised a mixture of two solvents: Solvent A (2% (v/v) acetonitrile containing 0.1% formic acid to enable excellent ionization in the APCI) and Solvent B (90% isopropanol containing 0.1% formic acid). Separation was achieved with a linear gradient of Solvent B from 30% to 90% in 12 min. In APCI**-**MS analysis, the temperature of the ion source was maintained at 100°C. Nitrogen desolvation gas was set at a flow rate of 500 l h^–1^ and the probe was heated to 400°C. Nitrogen served as the APCI nebulizer gas. The corona current was maintained at 20 µA, and the electron multiplier voltage was set to1700 eV. The parent scan was in the range of 50–500 *m*/*z*. The predicted elemental composition of individual intermediates was calculated using MassLynx™ Mass Spectrometry Software (Waters).

### Ultra-Performance Liquid Chromatography–Electrospray Ionization–High-Resolution Mass Spectrometry (UPLC-ESI-HRMS)

The ethyl acetate extractable samples and HPLC-purified intermediates were also analyzed using UPLC-ESI-HRMS. The separation conditions for UPLC were the same as those for UPLC-APCI-HRMS. Mass spectral data were collected in +ESI mode in separate runs on a Waters HDMS-QTOF synapt mass spectrometer operated in a scan mode from 50–500 *m*/*z*. The capillary voltage was set at 3000 V; the source and desolvation temperatures were 100°C and 250°C, respectively. The cone gas flow rate was 50 l h^−1^.

### NMR Spectroscopy

The ^1^H- and ^13^C-NMR spectra were recorded at 27°C using a Bruker AV600_GRC 600MHz NMR. Chemical shifts (δ) were recorded and shown as ppm values with deuterated methanol (99.8%, ^1^H: δ = 3.31 ppm; ^13^C: δ = 49.0 ppm) as the solvent and internal reference.

## Supporting Information

Figure S1 **High-resolution mass spectra of other ^13^C-labeled intermediates detected in ethyl-acetate extracts of** ***S. denitrificans*** **cells grown on [4C-^13^C]cholesterol (1 mM).** *The predicted elemental composition of individual intermediates was calculated using MassLynx™ Mass Spectrometry Software (Waters).
(TIF)

Figure S2 **High-resolution mass spectra of ^13^C-labeled intermediates detected in ethyl-acetate extracts of** ***S. denitrificans*** **cells grown on [2,3,4C-^13^C]testosterone (1 mM).** *The predicted elemental composition of individual intermediates was calculated using MassLynx™ Mass Spectrometry Software (Waters).
(TIF)

Figure S3 **ESI- (A) and (B) APCI-mass spectra of HPLC-purified compound 1.** The predicted elemental composition of the product ions was calculated using MassLynx™ Mass Spectrometry Software (Waters).
(TIF)

Figure S4 ^1^H-NMR (A), ^13^C-NMR (B), ^1^H-^1^H COSY (C), HMBC (D), and HSQC (E) spectra of HPLC-purified compound 1 (600 MHz, CD_3_OD).
(PDF)

Table S1 Steroid C26-hydroxylase activity was detected in *G. cholesterolivorans*, but not in *S. denitrificans* cells.
(DOC)

Table S2 ^1^H, ^13^C, COSY, and HMBC interpretations of compound **1** [δ in ppm, multi. (*J* in Hz)].
(PDF)

## References

[1] Johnson DF, Bennett RD, Heftmann E (1963) Cholesterol in Higher Plants. Science 140: 198–199. 17819841 10.1126/science.140.3563.198

[2] Kochhar SP (1983) Influence of processing on sterols of edible vegetable oils. Prog Lipid Res 22: 161–188. 6356149 10.1016/0163-7827(83)90008-5

[3] Weete JD (1989) Structure and function of sterols in fungi. Adv Lipid Res 23: 484–491.

[4] Weete JD, Abril M, Blackwell M (2010) Phylogenetic distribution of fungal sterols. PLoS One 5: e10899. 20526375 10.1371/journal.pone.0010899 PMC2878339

[5] Ismail W, Chiang YR (2011) Oxic and anoxic metabolism of steroids by bacteria. J Bioremed Biodegrad S1: 001 doi:–10.4172/2155–6199

[6] Kieslich K (1985) Microbial side-chain degradation of sterols. J Basic Microbiol 25: 461–474. 3903107 10.1002/jobm.3620250713

[7] Horinouchi M, Hayashi T, Kudo T (2012) Steroid degradation in *Comamonas testosteroni* . J Steroid Biochem Mol Biol 129: 4–14. 21056662 10.1016/j.jsbmb.2010.10.008

[8] Fernandes P, Cruz A, Angelova B, Pinheiro HM, Cabral JMS (2003) Microbial conversion of steroid compounds: recent developments. Enzyme Microb Tec 32: 688–705.

[9] Doukyu N (2009) Characteristics and biotechnological applications of microbial cholesterol oxidases. Appl Microbiol Biotechnol 83: 825–837. 19495743 10.1007/s00253-009-2059-8

[10] Tak JD (1942) On bacteria decomposing cholesterol. Antonie van Leeuwenhoek 8: 32–40.

[11] Whitmarsh JM (1964) Intermediates of microbiological metabolism of cholesterol. Biochem J 90: 23–24.

[12] Arima K, Nagasawa M, Bae M, Tamura G (1969) Microbial transformation of sterols. Part I. Decomposition of cholesterol by microorganisms. Agric Biol Chem 33: 1636–1634.

[13] Nagasawa M, Bae M, Tamura G, Arima K (1969) Microbial transformation of sterols. Part II. Cleavage of sterol side chains by microorganisms. Agric Biol Chem 33: 1644–1650.

[14] Nagasawa M, Watanabe N, Hashiba H, Murakami M, Bae M, et al (1970) Microbial transformation of sterols. Part V. Inhibitors of microbial degradation of cholesterol. Agric Biol Chem 34: 838–844.

[15] Owen RW, Mason AN, Bilton RF (1983) The degradation of cholesterol by *Pseudomonas* sp. NCIB 10590 under aerobic conditions. J Lipid Res 24: 1500–1511. 6655367

[16] Sih CJ, Tai HH, Tsong YY (1967) The mechanism of microbial conversion of cholesterol into 17-keto steroids. J Am Chem Soc 89: 1957–1958. 6040528 10.1021/ja00984a039

[17] Sih CJ, Tai HH, Tsong YY, Lee SS, Coombe RG (1968) Mechanisms of steroid oxidation by microorganisms. XIV. pathway of cholesterol side-chain degradation. Biochemistry 7: 808–818. 4296193 10.1021/bi00842a039

[18] Sih CJ, Wang KC, Gibson DT, Whitlock HWJ (1965) On the mechanism of ring A cleavage in the degradation of 9,10-seco steroids by microorganisms. J Am Chem Soc 87: 1386–1387. 14293760 10.1021/ja01084a043

[19] Sih CJ, Lee SS, Tsong YY, Wang KC (1965) 3,4-Dihydroxy-9,10-secoandrosta-1,3,5(10)-triene-9,17-dione. An intermediate in the microbiological degradation of ring A of androst-4-ene-3,17-dione. J Am Chem Soc 87: 1385–1386. 5908120

[20] Sih CJ, Lee SS, Tsong YY, Wang KC (1966) Mechanisms of steroid oxidation by microorganisms. VIII. 3,4-Dihydroxy-9,10-secoandrosta-1,3,5(10)-triene-9,17-dione, an intermediate in the microbiological degradation of ring A of androst-4-ene-3,17-dione. J Biol Chem 241: 540–550. 5908120

[21] Fahrbach M, Kuever J, Meinke R, Kämpfer P, Hollender J (2006) *Denitratisoma oestradiolicum* gen. nov., sp. nov., a 17beta-oestradiol-degrading, denitrifying betaproteobacterium. Int J Syst Evol Microbiol 56: 1547–1552. 16825628 10.1099/ijs.0.63672-0

[22] Horinouchi M, Hayashi T, Yamamoto T, Kudo T (2003) A new bacterial steroid degradation gene cluster in *Comamonas testosteroni* TA441 which consists of aromatic-compound degradation genes for seco-steroids and 3-ketosteroid dehydrogenase genes. Appl Environ Microbiol 69: 4421–4430. 12902225 10.1128/AEM.69.8.4421-4430.2003 PMC169130

[23] Van der Geize R, Yam K, Heuser T, Wilbrink MH, Hara H, et al (2007) A gene cluster encoding cholesterol catabolism in a soil actinomycete provides insight into *Mycobacterium tuberculosis* survival in macrophages. Proc Natl Acad Sci USA 104: 1947–1952. 17264217 10.1073/pnas.0605728104 PMC1794314

[24] Pandey AK, Sassetti CM (2008) Mycobacterial persistence requires the utilization of host cholesterol. Proc Natl Acad Sci USA 105: 4376–4380. 18334639 10.1073/pnas.0711159105 PMC2393810

[25] Van der Geize R, Hessels GI, van Gerwen R, van der Meijden P, Dijkhuizen L (2002) Molecular and functional characterization of kshA and kshB, encoding two components of 3-ketosteroid 9alpha-hydroxylase, a class IA monooxygenase, in *Rhodococcus erythropolis* strain SQ1. Mol Microbiol 45: 1007–1018. 12180920 10.1046/j.1365-2958.2002.03069.x

[26] Capyk JK, D’Angelo I, Strynadka NC, Eltis LD (2009) Characterization of 3-ketosteroid 9{alpha}-hydroxylase, a Rieske oxygenase in the cholesterol degradation pathway of *Mycobacterium tuberculosis* . J Biol Chem 284: 9937–9946. 19234303 10.1074/jbc.M900719200 PMC2665117

[27] Petrusma M, Dijkhuizen L, van der Geize R (2009) *Rhodococcus rhodochrous* DSM 43269 3-ketosteroid 9alpha-hydroxylase, a two-component iron-sulfur-containing monooxygenase with subtle steroid substrate specificity. Appl Environ Microbiol 75: 5300–5307. 19561185 10.1128/AEM.00066-09 PMC2725467

[28] Rosloniec KZ, Wilbrink MH, Capyk JK, Mohn WW, Ostendorf M, et al (2009) Cytochrome P450 125 (CYP125) catalyses C26-hydroxylation to initiate sterol side-chain degradation in *Rhodococcus jostii* RHA1. Mol Microbiol 74: 1031–1043. 19843222 10.1111/j.1365-2958.2009.06915.x PMC5218833

[29] Drzyzga O, Fernández de las Heras L, Morales V, Navarro Llorens JM, Perera J (2011) Cholesterol degradation by *Gordonia cholesterolivorans* . Appl Environ Microbiol 77: 4802–4810. 21622796 10.1128/AEM.05149-11 PMC3147395

[30] Dermer J, Fuchs G (2012) Molybdoenzyme that catalyzes the anaerobic hydroxylation of a tertiary carbon atom in the side chain of cholesterol. J Biol Chem 287: 36905–36916. 22942275 10.1074/jbc.M112.407304 PMC3481293

[31] Tarlera S, Denner EB (2003) *Sterolibacterium denitrificans* gen. nov., sp. nov., a novel cholesterol-oxidizing, denitrifying member of the beta-Proteobacteria. Int J Syst Evol Microbiol 53: 1085–1091. 12892131 10.1099/ijs.0.02039-0

[32] Chiang YR, Ismail W, Müller M, Fuchs G (2007) Initial steps in the anoxic metabolism of cholesterol by the denitrifying *Sterolibacterium denitrificans* . J Biol Chem 282: 13240–13249. 17307741 10.1074/jbc.M610963200

[33] Chiang YR, Ismail W, Heintz D, Schaeffer C, Van Dorsselaer A, et al (2008) Study of anoxic and oxic cholesterol metabolism by *Sterolibacterium denitrificans* . J Bacteriol 190: 905–914. 18039763 10.1128/JB.01525-07 PMC2223586

[34] Capyk JK, Kalscheuer R, Stewart GR, Liu J, Kwon H, et al (2009) Mycobacterial cytochrome p450 125 (cyp125) catalyzes the terminal hydroxylation of C_27_ steroids. J Biol Chem 284: 35534–35542. 19846551 10.1074/jbc.M109.072132 PMC2790983

[35] Hylemon PB, Harder J (1998) Biotransformation of monoterpenes, bile acids, and other isoprenoids in anaerobic ecosystems. FEMS Microbiol Rev 22: 475–488. 9990726 10.1111/j.1574-6976.1998.tb00382.x

[36] Heyen U, Harder J (2000) Geranic acid formation, an initial reaction of anaerobic monoterpene metabolism in denitrifying *Alcaligenes defragrans* . Appl Environ Microbiol 66: 3004–3009. 10877798 10.1128/aem.66.7.3004-3009.2000 PMC92103

[37] Brodkorb D, Gottschall M, Marmulla R, Lüddeke F, Harder J (2010) Linalool dehydratase-isomerase, a bifunctional enzyme in the anaerobic degradation of monoterpenes. J Biol Chem 285 30436–30442. 20663876 10.1074/jbc.M109.084244 PMC2945536

[38] Fuchs G, Boll M, Heider J (2011) Microbial degradation of aromatic compounds - from one strategy to four. Nat Rev Microbiol 9: 803–816. 21963803 10.1038/nrmicro2652

[39] Ismail W, Gescher J (2012) Epoxy coenzyme a thioester pathways for degradation of aromatic compounds. Appl Environ Microbiol 78: 5043–5051. 22582071 10.1128/AEM.00633-12 PMC3416408

[40] Wang PH, Leu YL, Ismail W, Tang SL, Tsai CY (2013) et?al (2013) The anaerobic and aerobic cleavage of the steroid core ring structure by *Steroidobacter denitrificans.* . J Lipid Res 54: 1493–1504. 23458847 10.1194/jlr.M034223 PMC3622341

[41] Leu YL, Wang PH, Shiao MS, Ismail W, Chiang YR (2011) A novel testosterone catabolic pathway in bacteria. J Bacteriol 193: 4447–4455. 21725000 10.1128/JB.00331-11 PMC3165539

[42] Pfenning N (1978) *Rhodocyclus purpureus* gen. nov. and sp. nov., a ring-shaped, vitamin B12-requiring member of the family Rhodospirillaceae. Int J Syst bacteriol 28: 283–288.

[43] Rabus R, Widdel F (1995) Anaerobic degradation of ethylbenzene and other aromatic hydrocarbons by new denitrifying bacteria. Arch Microbiol 163: 96–103. 7710331 10.1007/BF00381782

[44] Tschech A, Pfenning N (1984) Growth yield increase linked to caffeate reduction in *Acetobacterium woodii* . Arch Microbiol 137: 163–167.

