# Supplementary figures and images for: An Oxygenase-Independent Cholesterol Catabolic Pathway Operates under Oxic Conditions
Source: PLoS One. 2013 Jun 24;8(6):e66675. doi: 10.1371/journal.pone.0066675 (PMC3691188; doi:10.1371/journal.pone.0066675)

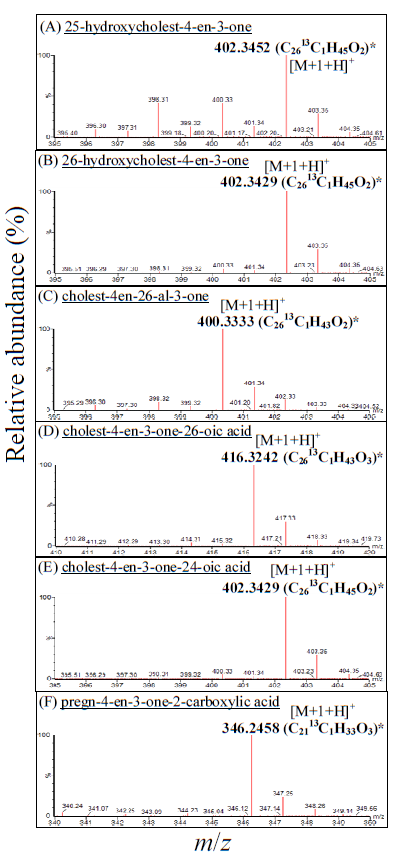

Supplement: Figure S1 — High-resolution mass spectra of other 13C-labeled intermediates detected in ethyl-acetate extracts of S. denitrificans cells grown on [4C-13C]cholesterol (1 mM). *The predicted elemental composition of individual intermediates was calculated using MassLynx™ Mass Spectrometry Software (Waters). (TIF) [file pone.0066675.s001.tif]

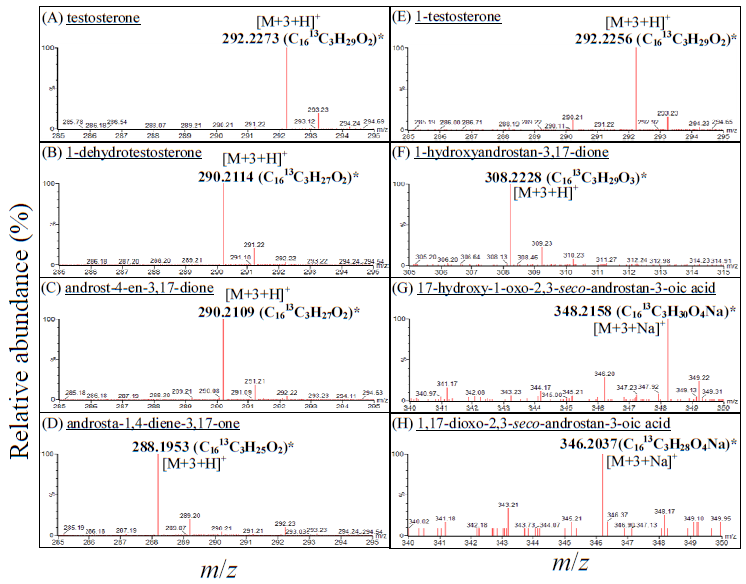

Supplement: Figure S2 — High-resolution mass spectra of 13C-labeled intermediates detected in ethyl-acetate extracts of S. denitrificans cells grown on [2,3,4C-13C]testosterone (1 mM). *The predicted elemental composition of individual intermediates was calculated using MassLynx™ Mass Spectrometry Software (Waters). (TIF) [file pone.0066675.s002.tif]

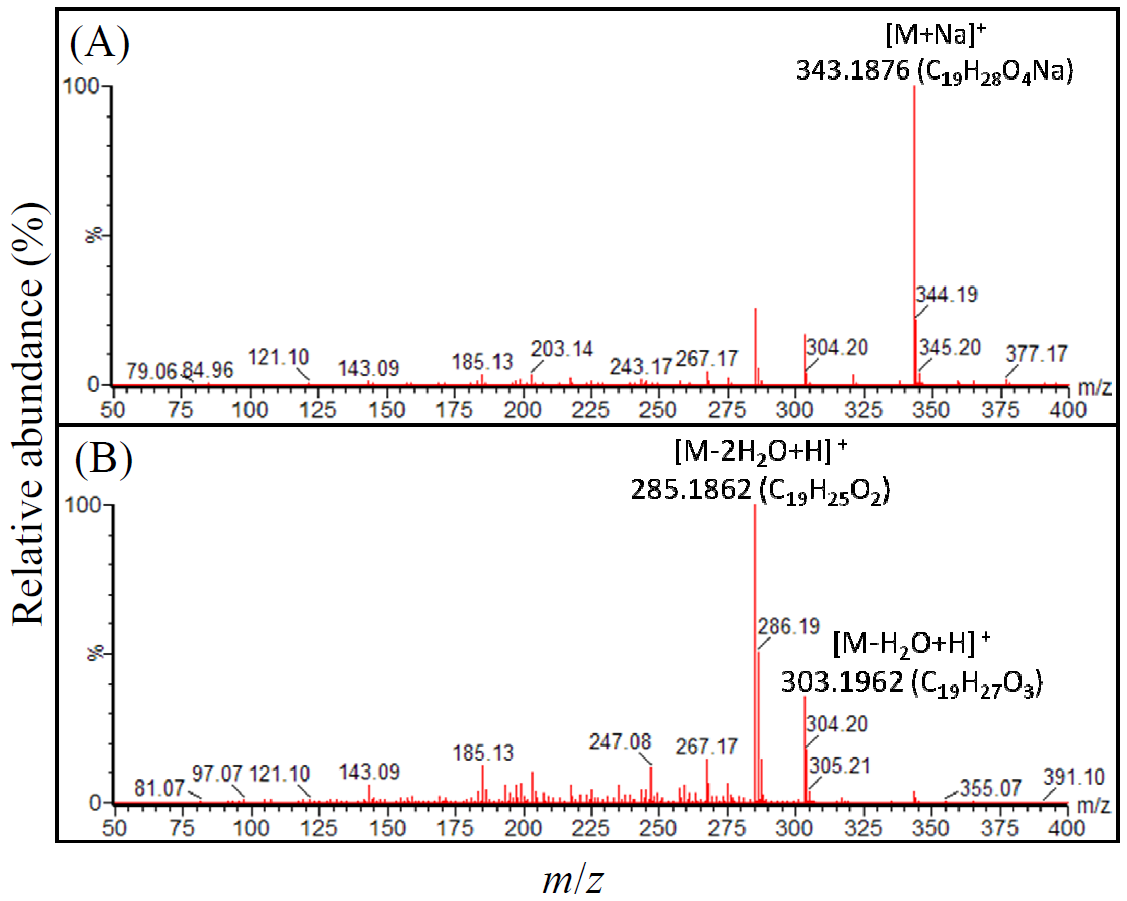

Supplement: Figure S3 — ESI- (A) and (B) APCI-mass spectra of HPLC-purified compound 1. The predicted elemental composition of the product ions was calculated using MassLynx™ Mass Spectrometry Software (Waters). (TIF) [file pone.0066675.s003.tif]

(A)

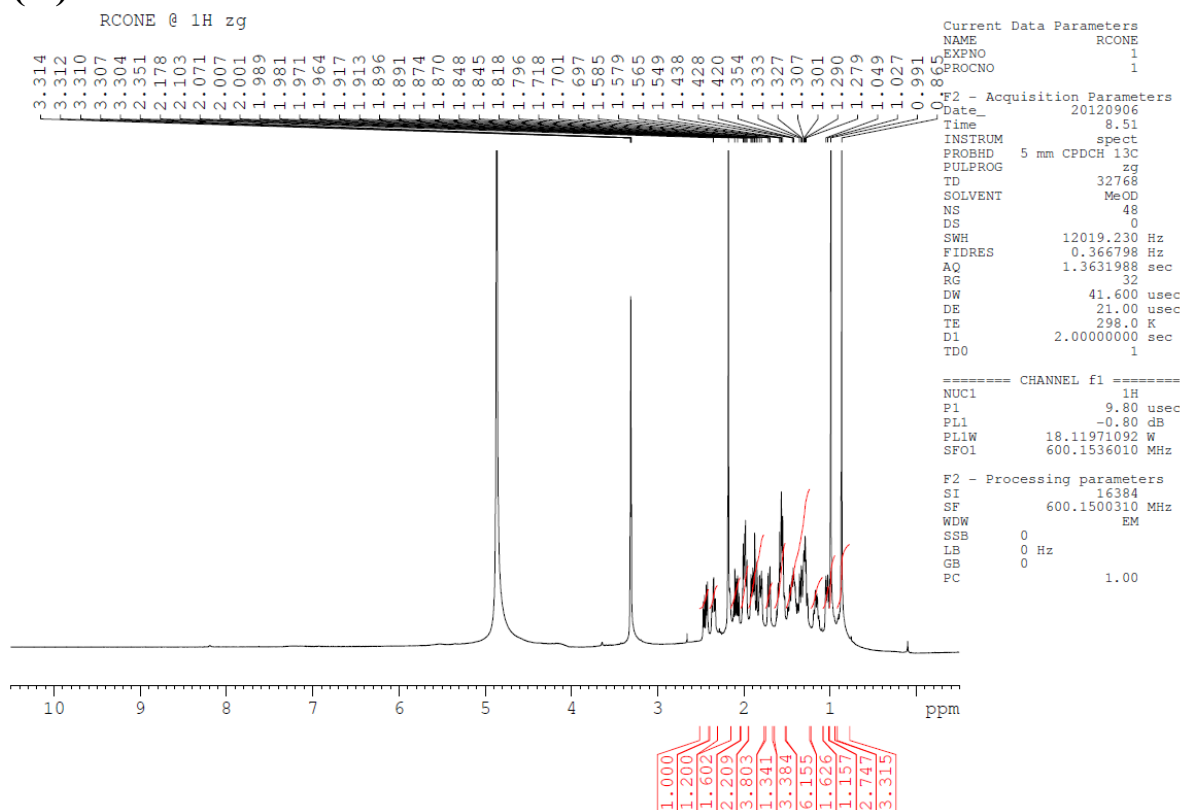

(B)

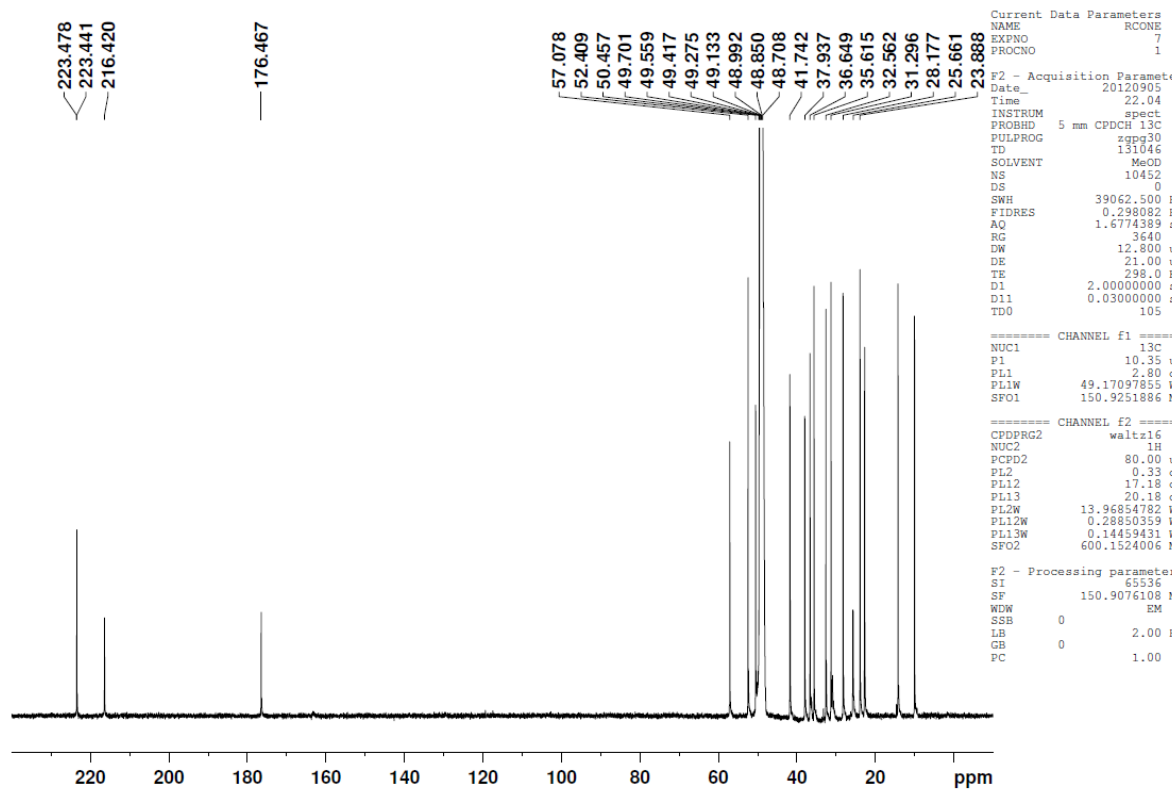

(C)

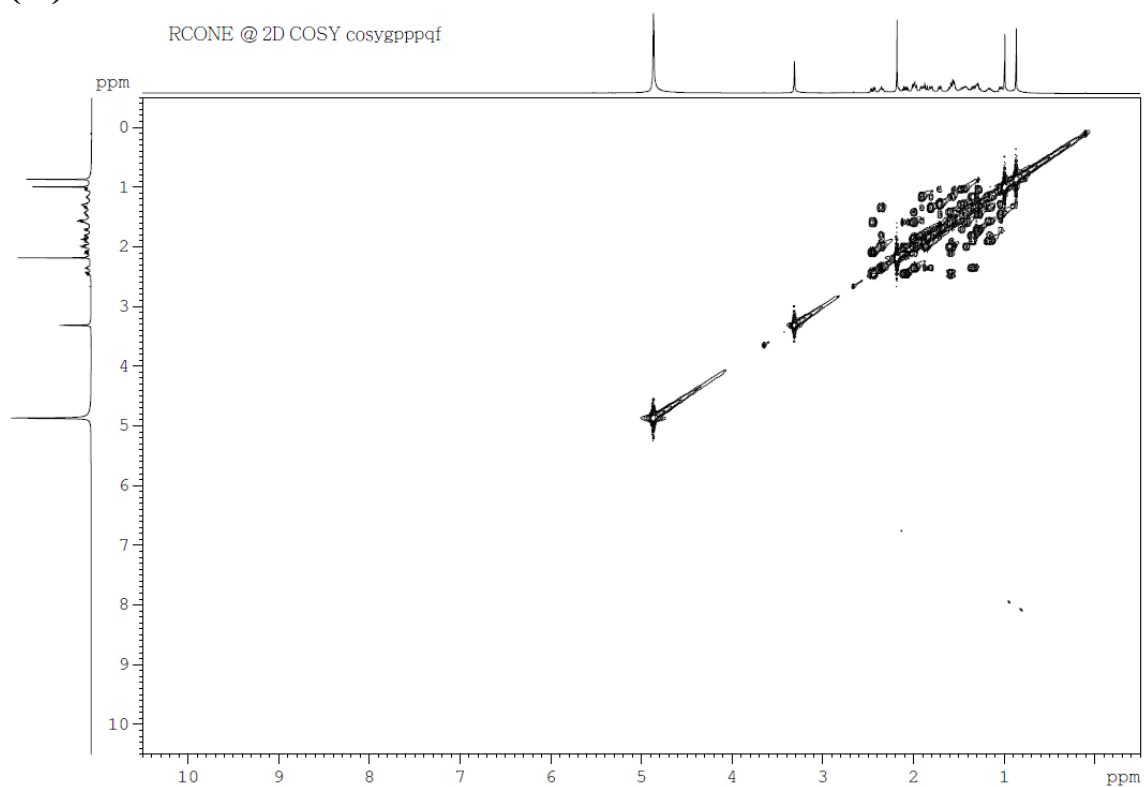

(D)

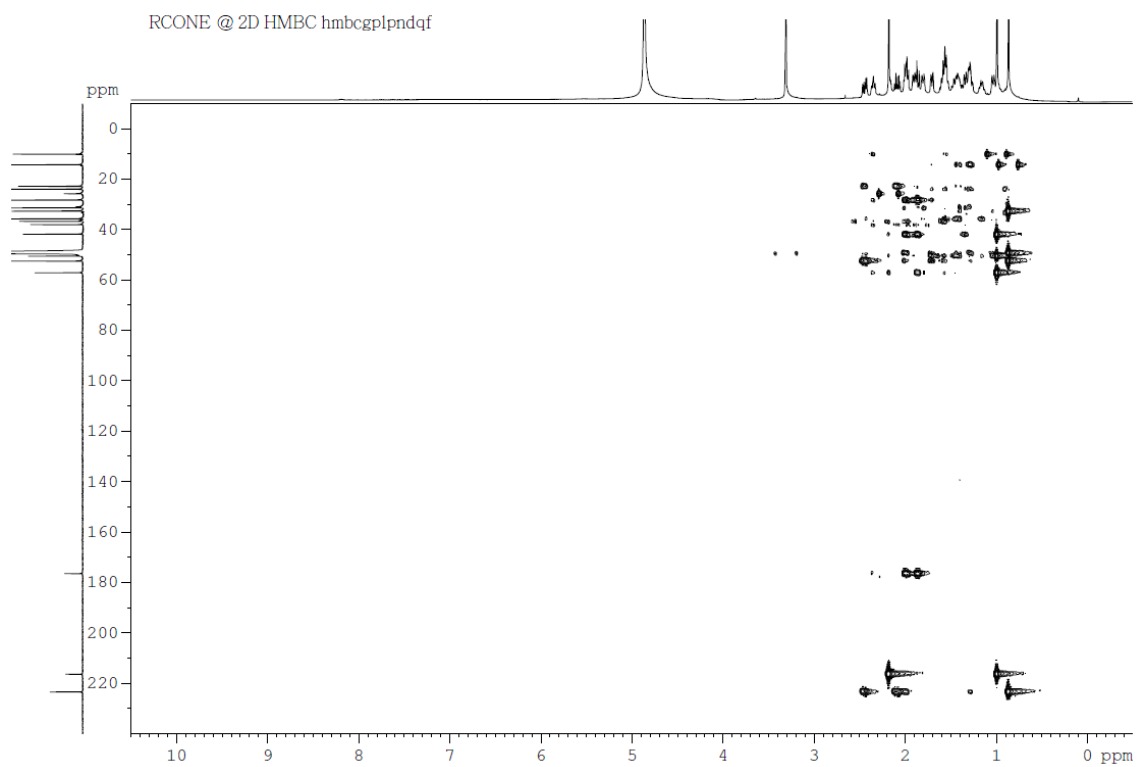

**(E)**

RCONE @ 2D HSQC-Echo hsqcetgpgsisp2.2 (most routine used)

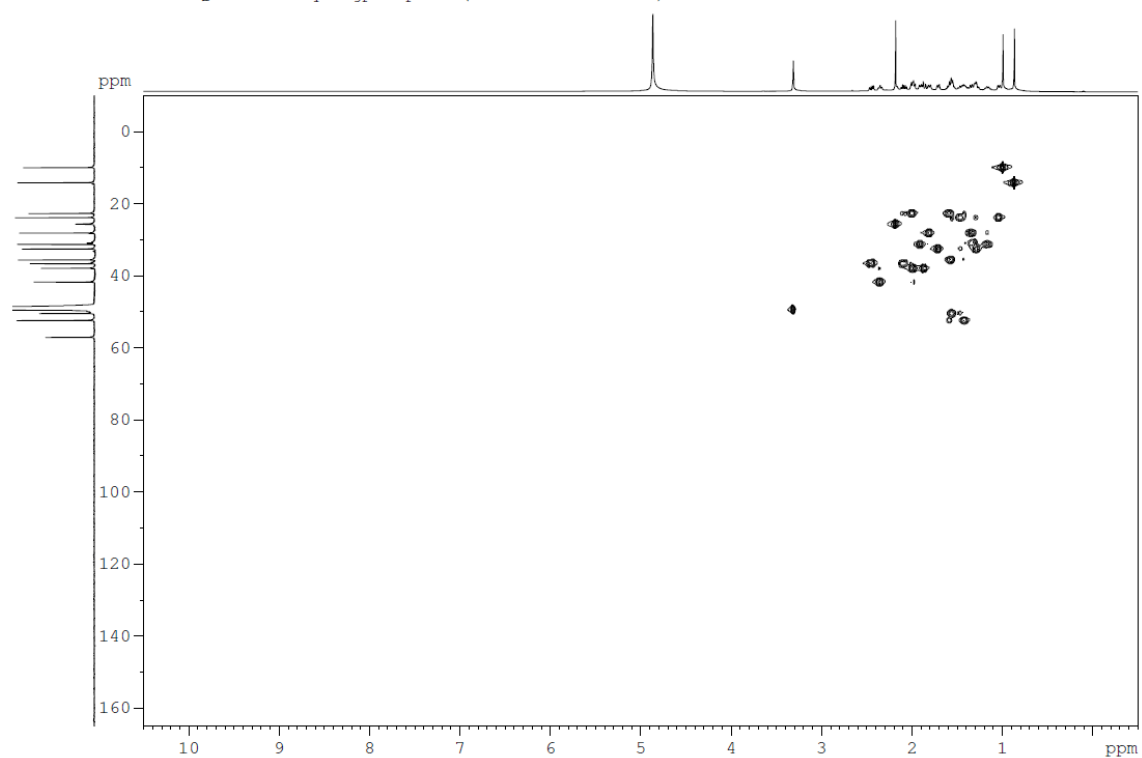

Supplement: Figure S4 — 1H-NMR (A), 13C-NMR (B), 1H-1H COSY (C), HMBC (D), and HSQC (E) spectra of HPLC-purified compound 1 (600 MHz, CD3OD). (PDF) [file pone.0066675.s004.pdf]
