# Supplementary material for: An Oxygenase-Independent Cholesterol Catabolic Pathway Operates under Oxic Conditions
Source: PLoS One. 2013 Jun 24;8(6):e66675. doi: 10.1371/journal.pone.0066675 (PMC3691188; doi:10.1371/journal.pone.0066675)
Supplement: Table S1 — Steroid C26-hydroxylase activity was detected in G. cholesterolivorans, but not in S. denitrificans cells. (DOC) [file pone.0066675.s005.doc]

**Table S1.** Steroid C26-hydroxylase activity was detected in *G. cholesterolivorans*, but not in *S. denitrificans* cells.

| Assay conditions | *G. cholesterolivorans* | *S. denitrificans* |
| --- | --- | --- |
| aerobic | + | - |
| strictly anaerobic | - | - |
