# Supplementary material for: An Oxygenase-Independent Cholesterol Catabolic Pathway Operates under Oxic Conditions
Source: PLoS One. 2013 Jun 24;8(6):e66675. doi: 10.1371/journal.pone.0066675 (PMC3691188; doi:10.1371/journal.pone.0066675)
Supplement: Table S2 — 1H, 13C, COSY, and HMBC interpretations of compound 1 [δ in ppm, multi. (J in Hz)]. (PDF) [file pone.0066675.s006.pdf]

Table S2. ^{1}H, ^{13}C, COSY, and HMBC interpretations of compound **1** [δ in ppm, multi. (*J* in Hz)].

position	**1**[a]			
	^{13}C [b]	^{1}H	COSY (H↔H)	HMBC (H→C)
1	216.4 s			
2	25.7 q	2.18 s		1, 10
3	176.5 s			
4^a	37.9 t	1.98 dd (15.0, 4.2)	4^b, 5	3, 5, 6
4^b		1.86 dd (15.0, 1.8)	4^a, 5	3, 5, 6, 10
5	41.7 d	2.36 m	4^a, 4^b, 6^a, 6^b	19
6^a	28.2 t	1.81 m	5, 6^b, 7^a	7
6^b		1.35 m	5, 6^a, 7^a, 7^b	5
7^a	31.3 t	1.91 m	6^b, 7^b, 8	5, 6
7^b		1.17 m	7^a, 6^a, 6^b, 8	8
8	35.6 d	1.57	7^a, 7^b	6, 9, 14
9	50.5 d	1.55	11^b	8, 11
10	57.1 s			
11^a	23.9 t	1.47 m	11^b, 12^a, 12^b	
11^b		1.04 m	9, 11^a, 12^a, 12^b	9
12^a	32.6 t	1.71 m	11^a, 11^b, 12^b	9, 14
12^b		1.28 m	11^a, 11^b, 12^a	11, 13, 17, 18
13	50.5 s			
14	52.4 d	1.42 m	15^a, 15^b	8, 9
15^a	22.7 t	1.60	15^b, 16^a, 16^b	17
15^b		2.00 m	15^a	14
16^a	36.7 t	2.45 dd (16.0, 7.5)	15^a, 16^b	14, 15, 17
16^b		2.08 dd (16.0, 7.5)	15^a, 16^a	15, 17
17	223.5 s			
18	14.2 q	0.87 s		12, 13, 14, 17
19	10.0 q	0.99 s		1, 5, 9, 10

[a] Signals without multiplicity were overlapped, and were picked up from the HSQC spectrum.
[b] Multiplicities were obtained from DEPT experiment.
